# Higher anthocyanin intake is associated with lower depressive symptoms in adults with and without major depressive disorder

**DOI:** 10.1002/fsn3.3850

**Published:** 2023-11-22

**Authors:** Annika Mestrom, Karen E. Charlton, Susan J. Thomas, Theresa A. Larkin, Karen L. Walton, Asmahan Elgellaie, Katherine Kent

**Affiliations:** ^1^ School of Medical, Indigenous and Health Sciences, Faculty of Science, Medicine and Health University of Wollongong Wollongong New South Wales Australia; ^2^ Graduate School of Medicine, Faculty of Science, Medicine and Health University of Wollongong Wollongong New South Wales Australia

**Keywords:** anthocyanins, depression, diet, major depressive disorder

## Abstract

**Background:**

Major depressive disorder (MDD) is a significant cause of disability globally and an emerging body of evidence suggests that dietary components, including flavonoids, may impact depression‐related biochemical pathways. Further research that characterizes dietary intake of flavonoids in diverse population groups, including people with MDD and explores the relationship between flavonoid intake and depression is needed. This study aimed to determine dietary flavonoid and subclass intake and assess the association with depressive symptomatology in a sample of adults with and without MDD.

**Methodology:**

Participants with and without MDD (determined using DSM 5) completed the Depression, Anxiety, and Stress Scale‐21 (DASS‐21). Diet history interviews were analyzed using *PhenolExplorer* to quantify flavonoid subclasses (flavan‐3‐ols, flavonols, anthocyanins, flavones, flavanones, isoflavones), and total flavonoid intake. Independent *t*‐tests and linear regression, adjusting for age, sex, and BMI were performed.

**Results:**

Participants (*n* = 93; 75% female) had a mean age of 26.0 ± 8.2 years. Participants with MDD had significantly higher DASS‐depression scores (*n* = 44; DASS‐depression 27.3 ± 9.8) compared to participants without MDD (*n* = 49; DASS‐depression 3.1 ± 4.4; *p* < .001). Intakes of total flavonoids and subclasses were similar between groups, except for anthocyanins where participants with MDD reported lower intakes of anthocyanins compared to participants without MDD (median intake: 0.08 mg/day and 11.6 mg/day, respectively; *p* = .02). In the total sample, higher anthocyanin intake was associated with lower DASS‐depression score (B = ‐4.1; SE = 1.8; 95% CI [−7.7, −0.4]; *p* = .029).

**Conclusion:**

Intake of total flavonoids and most subclasses were similar between people with and without MDD. However, a dietary deficit of anthocyanins (found in purple/red fruits and vegetables) was evident in participants with MDD, and higher anthocyanin intake was associated with lower depressive symptomatology in the total sample. Further research in larger samples is warranted to explore if the documented association is independent of MDD status.

## Graphical abstract

This study explored the association between flavonoid intake and depressive symptomatology in adults with and without major depressive disorder (MDD), finding that while total flavonoid intake and most subclasses were similar between groups, a dietary deficit of anthocyanins (found in purple/red fruits and vegetables) was evident in participants with MDD, and higher anthocyanin intake was associated with lower depressive symptomatology (Depression, Anxiety, and Stress Scale; DASS‐21).

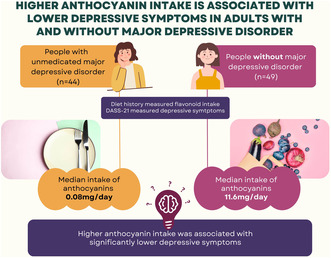

## 1 INTRODUCTION

Mood disorders are widespread and increasing, and a leading cause of disability globally (World Health Organization, 2017). Major depressive disorder (MDD) is estimated to affect more than 300 million adults worldwide (Global Burden of Disease Studies, 2016) with an increase of more than 25% during the SARS‐CoV‐2 pandemic (Santomauro et al., 2021). In Australia, depression was among the top three leading causes of years lived with disability in 2018 (Australian Institute of Health and Welfare, 2021). MDD is a complex disorder, distinguished by consistent low mood or anhedonia for a period of more than 2 weeks (*Diagnostic and statistical manual of mental disorders: DSM*‐5, 2013). Those living with MDD can also concurrently experience changes in appetite, low energy levels, sleep changes, difficulty concentrating or decision‐making, and significantly reduced quality of life (*Diagnostic and statistical manual of mental disorders: DSM*‐5, 2013). Moreover, subclinical depressive symptoms are highly prevalent, and associated with deficits in well‐being and functioning (Goldney et al., 2004). It is thought that the high prevalence of depression may be related, in part, to the more proinflammatory modern lifestyles, characterized by stress, sedentarism, and poor nutrition (Hidaka, 2012; Logan & Jacka, 2014). This, and the fact that depression only modestly responds to first‐line pharmaceutical and psychological therapies, explains why the disease burden attributable to mood disorders is predicted to escalate worldwide over the coming decades (Sarris et al., 2015; Whiteford et al., 2013). The identification of modifiable risk factors that may prevent or abate disease progression is essential to decrease the burden of depression (Firth et al., 2020).

Growing research suggests that lifestyle factors, including diet, play a significant mediating role in the development, progression, and treatment of MDD (Firth et al., 2020). This research has contributed to the rapidly emerging, interdisciplinary field of nutritional psychiatry, that integrates knowledge from nutrition science, psychology, neuroscience, and psychiatry to better understand how dietary choices impact brain function and mental well‐being. Research in this field aims to establish evidence‐based dietary recommendations for the prevention and management of mood disorders like MDD (Adan et al., 2019). Flavonoids are non‐nutritive compounds widely distributed in plants that are considered to be partly responsible for the neuroprotective effect of a fruit and vegetable‐rich diet (Ali et al., 2021). There are six major flavonoid subclasses, flavonols, flavones, flavan‐3‐ols, flavanones, anthocyanins, and isoflavones, that are found in fruits, grains, tea, wine and vegetable roots, stems, and leaves. Each subclass is found in a major dietary source, for example, citrus fruits are a major source of flavanones, whilst isoflavones are primarily found in soy products. Flavonoids have demonstrated compelling antidepressant properties in animal studies (Guan & Liu, 2016; Khan et al., 2018), but evidence in humans is less robust. A 2021 systematic review and meta‐analysis conducted by Ali et al. (Ali et al., 2021) noted that around half of the 46 included studies found that flavonoids had a significant and beneficial effect on depressive symptoms. However, many of the participants in the systematic review were older than 40 years of age and the primary measures of flavonoid exposure were isoflavones, a flavonoid subclass infrequently consumed in Australia (Kent et al., 2018). Other research demonstrates the potential mood benefits associated with consuming flavonoid‐rich foods. For example, goji berry consumption over 2 weeks increased mood in a randomized controlled trial (Amagase & Nance, 2008). Further, acute mood improvements have been reported in children and young adults following consumption of flavonoid‐rich blueberries (Khalid et al., 2017). Despite these promising results from intervention studies, there is a need for additional observational literature to explore the relationship between longer term flavonoid intake and depressive outcomes and compare differences in habitual intakes between people with and without MDD. Therefore, the aim of the study was to estimate the habitual intake of flavonoids and determine whether this is related to self‐reported depressive symptoms among adults with and without MDD. It was hypothesized that the diets of people with MDD would be lower in flavonoid content compared to people without MDD, and that higher flavonoid and subclass intakes would be associated with lower depressive symptoms.

## 2 MATERIALS AND METHODS

Data were collected in 2018 and 2019 as part of an ongoing, broader study that aims to examine the associations between mental health and physical health parameters in people with and without MDD. Details of the broader study have been previously published (Mills et al., 2018). Participants aged between 18 and 63 years were recruited via media and poster advertisements. To be eligible for inclusion in the MDD group, participants needed to meet the diagnostic criteria for MDD as determined by a Mini International Neuropsychiatric Interview (version 7.0.2) for DSM‐5 (Sheehan et al., 1998). Participants without MDD were generally healthy, had no current or previous psychiatric diagnoses, and were screened to confirm they did not meet the DSM‐5 criteria for MDD. Exclusion criteria across both groups were neurological, degenerative or substance use disorders, use of corticosteroid medication or psychotropic medications, including antidepressant medication, within the previous 2 months. Participants were also excluded if they had serious pre‐existing medical conditions. Participants provided voluntary written informed consent. The study was approved by the the University of Wollongong Human Research Ethics Committee (HREC2018/076).

Sociodemographic characteristics were obtained by questionnaire, and weight and height were measured to calculate BMI as weight (kg)/height (m)^2^. Participants also recorded lifestyle behaviors such as habitual hours of sleep and physical activity. Participants completed Depression and Anxiety Stress Scales‐21 (DASS‐21) (Lovibond & Lovibond, 1995), a validated psychometric tool (Henry & Crawford, 2005), which collects self‐reported psychological symptoms. The DASS‐21 measures psychological distress on *Depression*, *Anxiety*, and *Stress* subscales, with possible scores ranging from 0 to 21 for each. Higher scores for DASS‐21 Stress (DASS‐stress), Anxiety (DASS‐anxiety), and *Depression* (DASS‐depression) suggest greater symptom severity. Scores were doubled to be comparable to the original DASS‐42 (Lovibond & Lovibond, 1995). DASS‐depression scores >21 indicate severe levels of depressive symptoms (Lovibond & Lovibond, 1995).

Usual dietary intake was obtained via diet history interviews conducted by trained researchers. Participants used food models and measuring cups when estimating portion sizes to minimize recall bias. Dietary data was entered into the *Foodworks* (Version 10 professional, Xyris Software) dietary assessment program, informed by the AUSNUT 2013 nutrient database (AUSNUT 2011–13 Food Nutrient Database, 2013) to estimate energy intake. Dietary data were screened by an expert dietitian (KW) and excluded if the records were deemed to be incomplete or inaccurate, with insufficient detail to estimate nutrient intakes. The average daily intake of flavonoid subclasses was calculated by cross‐referencing plant‐based food items to the online Phenol‐Explorer database (Rothwell et al., 2013). If a food was not listed in the Phenol‐Explorer database, it was searched for in the US Department of Agriculture Database (USDA) for the Flavonoid Content of Selected Foods (Haytowitz et al., 2018). Foods not found in either database were assumed to have zero flavonoid content. The flavonoid contribution from each food was calculated by multiplying the content values retrieved from Phenol‐Explorer (mg) by the daily average consumption of each food (g). Retention factors were used where possible to account for cooking and processing methods. The food contribution values were summed to estimate daily intake of each flavonoid subclass for each participant and to assess total overall flavonoid intake.

### 2.1 Statistical analyses

All statistical analyses were conducted using SPSS (version 25 SPSS Statistic Subscription, IBM, Chicago, IL, USA 2019). Statistical significance was accepted at alpha *p* < .05. Descriptive statistics were used to report the study sample and group demographics (e.g., proportions for categorical variables; means with standard deviations and/or medians and interquartile ranges for continuous variables). Percentage contribution of individual foods to intake of flavonoids subclasses was calculated. A Shapiro–Wilk test assessed variables for normality. Flavonoid and subclass intake variables were not normally distributed and were, therefore, log transformed. Independent *t*‐tests compared differences in flavonoid and subclass intake between groups. A series of linear regression models (univariate and multivariate models controlling for age, sex, and BMI) were conducted to examine the association between intake of total flavonoids and each of the flavonoid subclasses with DASS‐depression score. Power calculations performed using G*Power indicated that for linear multiple regression, with four predictors, a medium effect size, and probability of .05, a sample size of 85 participants is needed to achieve .80 power (Faul et al., 2009).

## 3 RESULTS

The characteristics of study participants are shown in Table 1. Of recruited participants (*n* = 123), 93 individuals had valid dietary data (44 with MDD [mean age 25.6 ± 6.3] and 49 without MDD [mean age 26 ± 10 years]), with no significant age difference between groups. There was no significant difference in sex distribution between groups with most participants being female (77% of participants with MDD and 73% without MDD). Participants with MDD had a significantly higher body mass index (BMI) than people without MDD but there was no difference in mean daily energy (kJ) intake between groups. This might be explained by significantly higher levels of physical activity in people without MDD. The mean DASS‐depression score for the total sample was 14.6 ± 14.2 and was significantly higher in participants with MDD (27.3 ± 9.8) compared with participants without MDD (3.1 ± 4.4). The mean DASS‐anxiety score in the MDD group was significantly higher than those without MDD (19.9 ± 9.05 and 3.2 ± 4.5, respectively). Lastly, the mean DASS‐stress score in those with MDD (25.3 ± 9.0) was significantly higher than those without MDD (4.9 ± 5.4).

**TABLE 1:** Comparison of demographic characteristics in participants with and without major depressive disorder (MDD).

	Total sample	Participants with MDD	Participants without MDD	*p*‐value
*N*	93	44	49	
Mean age (years), mean ± SD	26.0 ± 8.2	25.6 ± 6.3	26.4 ± 9.8	.631
Females, *n* (%)	70 (75%)	34 (77%)	36 (73%)	.675
BMI (kg/m^2^), mean ± SD	25.5 ± 6.1	27.0 ± 6.7	22.8 ± 3.8	<.001
Daily energy intake (KJ), mean ± SD	9546 ± 3797	9967.5 ± 4939.9	9104.7 ± 2407.8	.140
DASS‐depression score; mean ± SD	14.6 ± 14.2	27.3 ± 9.8	3.1 ± 4.4	<.001
DASS‐anxiety score; mean ± SD	11.1 ± 10.9	19.9 ± 9.05	3.2 ± 4.5	<.001
DASS‐stress score; mean ± SD	14.6 ± 12.6	25.3 ± 9.0	4.9 ± 5.4	<.001
Average sleep (hours)	7.2 ± 1.4	6.9 ± 1.6	7.5 ± 1.0	.022
Active hours per week	6.2 ± 3.5	5.1 ± 3.1	7.3 ± 3.6	.003

*Note*: *p*‐values for continuous variables derived from independent *t*‐test and chi‐square statistic for categorical variables.

Abbreviation: SD, Standard Deviation.

Median intake of total flavonoids was 174 mg/day, with no significant difference in total flavonoid intake evident between participants with and without MDD (Table 2). Flavan‐3‐ols and flavonols were the predominant flavonoid subclasses consumed. Oranges (60%) were the major contributor to flavanone intake and to flavone intake (40%). Soy milk was the predominant source of isoflavones. Tea (black 69% and green 15%) was the main source of flavan‐3‐ols and contributed to flavonol intake (black tea 16%). Blueberries (27%) and strawberries (17%) were top contributors to anthocyanin intake, followed by eggplant (17%). There was no significant difference in intake of flavonoid subclasses evident between groups except for anthocyanins, where participants with MDD (median intake 0.08 mg/day) had significantly lower intake compared with participants without MDD (median intake 11.6 mg/day), with a medium effect size. Linear regression models, both unadjusted and adjusted for age, sex, and BMI found a significant relationship between DASS‐depression total scores and anthocyanin intake (Table 3). No relationship was found between DASS‐depression scores and intake of the other subclasses, or total flavonoid intake.

**TABLE 2:** Comparison of flavonoids and subclass intakes (mg/day) in a sample of participants with and without major depressive disorder (MDD).

	Total sample (*n* = 93)		With MDD (*n* = 44)		Without MDD (*n* = 49)		*p*‐value and Cohen's D effect size*	
mg/day	Mean ± SD	Median (IQR)	Mean ± SD	Median (IQR)	Mean ± SD	Median (IQR)		
Total flavonoid intake	215.7 ± 216.6	173.7 (37.0–346.1)	220.4 ± 237.8	196.4 (45.5–273.9)	211.6 ± 198.1	169.4 (30.4–351.7)	.884	.037
Anthocyanin intake	23.6 ± 43.9	1.3 (0.0–23.8)	12.5 ± 31.2	11.6 (0.08–41.5)	33.6 ± 51.0	0.08 (0.0–11.2)	.002	.635
Flavan‐3‐ol intake	117.5 ± 183.0	38.4 (3.7–186.9)	135.8 ± 222.4	55.9 (3.4–187.5)	101.1 ± 139.0	28.2 (3.9–189.8)	.292	−.098
Flavanone intake	11.3 ± 28.4	0.9 (0.1–6.0)	11.8 ± 26.4	0.6 (0.03–11.5)	10.9 ± 30.3	1.2 (0.2–3.4)	.988	−.048
Flavone intake	1.5 ± 2.5	0.6 (0.2–1.5)	1.2 ± 2.0	0.4 (0.2–1.2)	1.8 ± 2.8	0.9 (0.4–2.1)	.339	.351
Flavonol intake	19.3 ± 18.4	14.1 (5.9–26.5)	18.0 ± 19.2	12.1 (4.4–25.6)	20.4 ± 17.8	14.9 (6.4–28.8)	.854	.291
Isoflavone intake	42.5 ± 122.0	0.0 (0.0–0.7)	41.0 ± 110.0	0.0 (0.0–1.8)	43.8 ± 133.0	0.07 (0.0–0.6)	.770	−.017

* *p*‐value and Cohen's D effect size derived from independent *t*‐test on log‐transformed data.

Abbreviations: IQR, Interquartile Range (25th–75th percentile); and SD, Standard Deviation.

**TABLE 3:** Linear regression analysis (unadjusted and adjusted for age, sex, and BMI) of association between DASS‐depression score and flavonoid/flavonoid subclass intake in a sample of participants with and without major depressive disorder (MDD).

	Unadjusted				Adjusted for age, sex, and BMI			
	B	SE	95% CI	*p*‐value	B	SE	95% CI	*p*‐value
Total flavonoid intake	−2.4	2.4	[−7.1, 2.4]	.320	−3.5	2.4	[−8.3, 1.3]	.151
Anthocyanin intake	−6.1	1.8	[−9.7, −2.6]	.001	−4.1	1.8	[−7.7, −0.4]	.029
Flavan‐3‐ol intake	0.01	1.8	[−3.5, 3.5]	.993	−2.6	1.8	[−6.2, 0.9]	.144
Flavanone intake	−0.4	2.5	[−5.2, 4.5]	.885	2.5	2.5	[−2.5, 7.5]	.324
Flavone intake	−8.8	5.4	[−19.5, 1.9]	.104	−6.9	5.3	[−17.5, 3.8]	.201
Flavonol intake	−6.1	3.5	[−13.1, 0.8]	.182	−5.5	3.7	[−12.7, 1.8]	.140
Isoflavone intake	−1.1	1.7	[−4.6, 2.3]	.512	0.4	1.6	[−2.8, 3.6]	.802

*Note*: *p*‐value derived from linear regression.

Abbreviations: 95% CI, 95% Confidence Interval; B, β Coefficient; SE, Standard Error.

## 4 DISCUSSION

The results of this study suggest that habitual intake of total flavonoids and most subclasses were similar between people with and without MDD; however, a dietary deficit of anthocyanins (compounds that provide the purple/red pigmentation in fruits and vegetables) was evident for participants with MDD, with a medium effect size. An inverse relationship between anthocyanin intake and depressive symptoms was found, where higher anthocyanin intake was associated with lower depressive symptomatology. Therefore, the hypothesis that the diets of people with MDD will be lower in flavonoid content compared to people without MDD was accepted for the anthocyanins subclass only. Additionally, the hypothesis that higher consumption of flavonoids would be associated with lower depressive symptoms was accepted in the anthocyanins subclass only.

Participants in our study sample, who were predominantly younger adults, reported consuming substantially lower amounts of flavonoids than the average Australian adult (mean = 454 mg/day) (Johannot & Somerset, 2006). Lower flavonoid intake in the current study could potentially be explained by lower levels of flavonoid‐rich tea consumed by young adults compared to middle‐aged and older adults in some Western countries (Johannot & Somerset, 2006). For example, one study in older Australian adults reported that black tea contributed around 95% of flavan‐3‐ol intake and 69% of flavonol intake in older adults, which is a much higher relative contribution than found in our study (Kent et al., 2018).

Our results showing the relationship between anthocyanins and depressive symptoms are similar to the Mediterranean Healthy Eating, Lifestyle, and Aging (MEAL) study which reported that higher intake of anthocyanins was associated with lower depression severity in a dose‐dependent manner (Godos et al., 2018). Interestingly, the MEAL study also found that total polyphenol intake was not associated with depression outcomes. Another study in middle‐aged Korean females found that intake of all flavonoid subclasses was lower in people with depression and that anthocyanins were inversely associated with depressive symptoms (Park et al., 2021). Conversely, anthocyanin intake was not associated with depressive outcomes in the all‐female Nurses' Health Study and Nurse's Health Study II cohorts. Instead, a greater intake of flavonol, flavone, and flavanone intake was associated with decreased risk of symptoms of depression (Chang et al., 2016), which was not found in our study. The role of anthocyanins in regulating mood has also been explored in intervention studies. A recent randomized, double‐blind, placebo‐controlled trial reported that adolescents whose diet was supplemented with a daily anthocyanin‐rich blueberry drink reported significantly fewer symptoms of depression than the placebo group after a period of 4 weeks (Fisk et al., 2020).

There are several proposed mechanistic pathways for how anthocyanins may modulate MDD. Anthocyanins have been shown to oppose the neuroinflammatory environment thought to underlie depression by inhibiting the activity of inflammatory species (Leonard, 2018; Rafael et al., 2019). The antioxidant capacity of anthocyanins may neutralize free radicals and protect against oxidative stress, serving to increase cerebral blood flow to regions related to emotional modulation and cognitive control (Khalid et al., 2017). Anthocyanin compounds have also been shown to inhibit monoamine oxidase, which is involved in the oxidation of mood‐related neurotransmitters such as serotonin, dopamine, and noradrenaline (Watson et al., 2015). Anthocyanins and other flavonoids have been shown to bind to GABA_A_ receptors, a class of receptors responsible for controlling fear, stress, and anxiety in the central nervous system (Rafael et al., 2019; Youdim et al., 2004). Lastly, it has been proposed that anthocyanin‐rich foods impact gut microbiota composition and encourage antidepressant pathways via the gut–brain axis such as improving blood–brain barrier integrity, regulating neuroinflammation, and promoting neurotransmitter production (Zhong et al., 2023). These proposed mechanisms highlight the diverse ways in which anthocyanins may impact MDD. To establish their precise involvement, further research is needed to confirm which pathways play a significant role in MDD development and treatment.

In our study, estimated energy intake was similar between groups, but BMI was higher in participants with MDD compared to those without MDD. This indicates the potential role for other factors, such as exercise in influencing the relationship between diet and depression. Indeed, participants with MDD engaged in less physical activity than participants without MDD. Previous studies have demonstrated a moderate effect of exercise on depression reduction compared to control interventions (Cooney et al., 2013). Considering this, it is recommended for exercise to be a controlled variable in future studies with adequate power. It is also possible that overall dietary quality (not considered in our study) has a role in influencing depression, and as such there is a growing movement toward investigating the effect of global dietary patterns, rather than discrete nutrients, on disease outcomes. Various observational studies have associated plant‐rich eating patterns that are low in discretionary food items, such as a Mediterranean‐style diet, with reduced prevalence of, and risk for, depression (Lai et al., 2014; Psaltopoulou et al., 2013). However, as our study groups showed similar energy intake, intake of other flavonoid subclasses suggests similar consumption of plant foods across participants with and without MDD. This suggests the potential for anthocyanins to act independently on antidepressant biological pathways that drive the neuroprotective effects of plant‐rich eating patterns. If confirmed, this finding has valuable implications for the field of nutritional psychiatry as identifying bioactive compounds may help to illuminate the dynamics of depressive pathophysiology and elucidate the possibility of dietary and nutraceutical prescriptions.

This study provides a novel contribution to the emerging body of evidence about the role of dietary components in mood disorders. The strength of the study is recruitment of adults with untreated MDD, which is a previously under‐researched group. Objective measures of flavonoid intake, such as biomarkers, are challenging to assess due to the complexity of flavonoid metabolism and the absence of universally accepted biomarkers for all flavonoid subclasses, making dietary assessment the most practical and comprehensive approach to estimating flavonoid intake. Diet histories, used in this study, provide a measure of habitual dietary intake, important to overcome issues with short dietary assessment methods for measuring flavonoids (Kent et al., 2018). Further research is needed to characterize biomarkers of flavonoid intake (all subclasses) to assist in elucidating potentially causative mechanisms. The study also has some limitations. Firstly, the cross‐sectional design does not have the ability to establish causal relationship, and depression and diet may exhibit bidirectional causality. The sample size in the present study limits our ability to explore the relationship between flavonoid and subclass intake within subgroups, and further research in larger samples is warranted to explore the relationship between anthocyanin intake and DASS‐depression scores within a sample of people with untreated MDD only. While, in our study, sex differences in the intake of flavonoids or depressive outcomes were not found, other studies have reported that sex is a significant predictor of some disordered eating behaviors that could impact the intake of dietary flavonoids (Mills et al., 2018). Diet history interviews, whilst considered more comprehensive than some other methodologies, do present a risk for recall and social desirability biases (Martin et al., 2002). In terms of flavonoid estimation, assumptions had to be made where complex foods were recorded in diet histories (e.g., ready‐to‐eat commercial meals). In many instances where recipes were unclear, flavonoid intake was assumed to be zero. Therefore, flavonoid estimates are likely to underrepresent true consumption. As in other Australian studies, a lack of locally representative flavonoid content values may also reduce the accuracy of flavonoid intake estimates (Johannot & Somerset, 2006). Another limitation of this study is the absence of data on participants’ income, education level, and socioeconomic status as previous research indicated that these factors may influence an individual's dietary choices, particularly for fruits and vegetables (Darmon & Drewnowski, 2008).

In conclusion, among mostly young adults with and without major depressive disorder, dietary intakes higher in anthocyanins were associated with lower depressive symptoms. This research contributes to the growing evidence base of nutritional psychiatry through novel findings about the potential role of dietary anthocyanins for mental well‐being, in addition to their potential role in influencing the mechanistic pathways of MDD. Further research to extend these findings is warranted, including research that explores the relationship of specific anthocyanin‐rich foods with cognitive and mood outcomes, and further understanding of dose–response relationships. This research will provide a better understanding of how diet affects mood in people with MDD, so that future nutrition therapies can be used to complement existing treatment plans, promote overall health, and reduce potential comorbidities in this vulnerable group.

## AUTHOR CONTRIBUTIONS

**Annika Mestrom:** Conceptualization (equal); data curation (equal); formal analysis (supporting); methodology (supporting); writing – original draft (equal). **Karen E. Charlton:** Conceptualization (equal); methodology (equal); project administration (equal); supervision (equal); writing – review and editing (equal). **Susan A. Thomas:** Conceptualization (supporting); data curation (supporting); formal analysis (supporting); funding acquisition (lead); methodology (equal); project administration (equal); resources (equal); supervision (equal); writing – review and editing (equal). **Theresa J. Larkin:** Conceptualization (supporting); funding acquisition (supporting); investigation (supporting); methodology (supporting); project administration (supporting); writing – review and editing (equal). **Karen L. Walton:** Conceptualization (supporting); funding acquisition (supporting); methodology (supporting); project administration (supporting); resources (equal); supervision (supporting); writing – review and editing (equal). **Asmahan Elgellaie:** Investigation (supporting); methodology (supporting); project administration (supporting); writing – review and editing (supporting). **Katherine Kent:** Conceptualization (supporting); formal analysis (lead); supervision (equal); writing – original draft (equal); writing – review and editing (lead).

## FUNDING INFORMATION

Funding for this study was provided by the University of Wollongong Australia.

## CONFLICT OF INTEREST STATEMENT

No authors have any competing financial interests in relation to the work described.

## ETHICS STATEMENT

Participants provided voluntary written informed consent. The study was approved by the University of Wollongong Human Research Ethics Committee (HREC2018/076).

## Data Availability

The data that support the findings of this study are available on request from the corresponding author.
